# Adolescent and Young Adult Testicular Germ Cell Tumors: Special Considerations

**DOI:** 10.1155/2018/2375176

**Published:** 2018-01-31

**Authors:** Amanda F. Saltzman, Nicholas G. Cost

**Affiliations:** Department of Surgery, Division of Urology, University of Colorado School of Medicine and Children's Hospital Colorado, Aurora, CO, USA

## Abstract

While testicular germ cell tumors (T-GCTs) make up only 0.5% of pediatric malignancies and less than 2% of adult malignancies, they comprise 14% of adolescent malignancies, making it the most common solid tumor in this age group. The transition in incidence at this age is also accompanied by a transition in tumor histology with adolescents having mostly pure embryonal carcinoma and mixed nonseminomatous germ cell tumors. Similar to T-GCTs of all ages, surgical excision with orchiectomy is the standard initial step in treatment. Chemotherapy, retroperitoneal lymph node dissection, and targeted treatment of distant metastases make even widely disseminated disease treatable and curable. For this reason, in many ways, the future focus has expanded beyond survival alone to emphasize quality of life issues such as fertility and hypogonadism. However, adolescents remain the age group least studied or understood as they fall in between the ages included in most study designs. Also, they require the most psychosocial support because of the challenges unique to the adolescent period. In this review, we aim to highlight the known outcome data for T-GCTs in this population and also to discuss the unique aspects of treatment and support for this age group.

## 1. Introduction

In pediatric oncology, significant advances have been made in survival of a variety of malignancies. The OS of children with cancer as a group approaches 80%, largely due to the collaborative efforts of cooperative groups [1]. However, numerous reports have shown that this triumph has not been evenly distributed across patients of all ages—the adolescent age group has not enjoyed the same success as younger children, and this has been specifically demonstrated for T-GCTs [2]. In fact, cancer in those aged 15–29 years kills more patients than any disease except suicide [3]. This is due to a host of reasons: delayed presentation and diagnosis [4], transition between adult and pediatric providers which may limit access to care [5], the disproportionate presence of high-risk pathologic components [6], poor treatment compliance [7], and a paucity of clinical trials and research focused on adolescents specifically. Perhaps the most relevant, however, is the lack of awareness of this age group as being unique [3].

Often, we assess adolescents along with those just slightly older and consider this group as adolescents and young adults (AYAs). The AYA population with cancer is a vulnerable group [4]. Compared with older adults with testicular cancer specifically, survival patterns differ [8], there are insurance coverage issues [5], and these patients are less likely to participate in clinical trials, are more likely to experience delays in diagnosis or treatment [9], and are more likely to suffer psychosocial problems and decreased quality of life related to their diagnosis [10, 11]. Because of these disparities, in 2006, the American Cancer Society and National Cancer Institute with help from the LIVESTRONG Young Adult Alliance called for future research to focus on cancer outcomes in AYA patients and established the AYA Oncology Progress Review Group [12]. Similarly, COG and SWOG have established dedicated AYA committees, and recently, the Society for Adolescent and Young Adult Oncology was funded. It is very important to understand that this term is used differently between studies and between large study groups: SEER 15–29 yr, NCI's AYA Oncology Progress Review Group 15–39 yr, and NCCN guideline 15–39 yr [13].

To properly develop a focus on AYA testicular cancer, there needs to be focus beyond just diagnosis and treatment—and must include a focus on fertility preservation, emotional support for patients and families, socioeconomic support, educational encouragement, palliative care, and survivorship specialists to meet the needs of this unique population [5]. There also must be collaboration and cooperation of providers who care for children and adults and fluid transition between the two.

## 2. Disease-Specific Aspects Unique to the AYA Population

### 2.1. Ethnicity

Testicular cancer is the most common urologic cancer in the AYA males [4, 13, 14]. Worldwide, it disproportionately affects men living in developed nations (USA, Canada, Denmark, Switzerland, Norway, Australia, New Zealand, etc.), where its incidence is attenuating over recent generations. In contrast, the incidence is rapidly increasing in countries undergoing developmental transition (Croatia, Slovenia, Singapore, the Philippines, China, Costa Rica, etc.) [15]. Testis cancer in general, disproportionately affects white men. While the incidence of testis cancer in AYAs is increasing overall, there has been a very large (58%) increase in the Hispanic population over the non-Hispanic white population (7%) [16]. Also, within the AYA group, in the adolescent population specifically, there appears to be a disproportionate population of Hispanic males affected [17]. While survival after testicular cancer is high, several large population-based studies of men with testicular cancer of all ages have shown that non-Hispanic whites have an increased OS when compared to Hispanic whites [18], African Americans [17, 19], and nonwhites [20]. This has been confirmed in the AYA population, with African Americans and Hispanics having worse OS and CSS than whites, even after adjustment for neighborhood socioeconomic status [21]. When looking at patients with pure seminoma, however, the racial disparities are less impactful for unclear reasons [21].

### 2.2. Neighborhood/Socioeconomic Status

A recent study using the California Cancer Registry examined the association between the patients' sociodemographic factors (race/ethnicity and neighborhood socioeconomic status) and survival of AYAs with testicular cancer from 1998 to 2000 [21]. They identified just over 14,000 patients and found that AYAs from middle and low socioeconomic neighborhoods had a much lower OS and cancer-specific survival than AYAs from high socioeconomic neighborhoods, even when controlling for race/ethnicity. This difference was seen in both patients with seminoma and those with nonseminoma [21]. This trend, worse outcomes in lower socioeconomic neighborhoods, has been well described in the oncologic literature, across various cancers and in both children and adults [17, 22, 23]. It has been suggested that neighborhood socioeconomic status is an independent risk factor for survival, not just a surrogate for individual socioeconomic status, and it mediates poorer outcomes through neighborhood level factors, such as social environment, reduced quality and availability of healthcare and support services, and chronic stress [21].

### 2.3. Age

Patients aged 15–24 years with T-GCTs had an improved OS, but not cancer-specific survival, than those aged 25–39 years [21]. However, when comparing patients aged 15–19 years to those <15 years, there are significant decreases in OS [1]. When examining event free survival (EFS), patients aged 13–19 years have been shown to have a 3-year EFS of 60%, significantly worse than patients aged < 13 years (87%) and patients aged >19 years (80%) [2]. The seeming contradictions of these studies are apparent; however, it is very important to identify the endpoint specified, because a disease like T-GCTs is very salvageable, even after metastasis and recurrence. Thus, differences in OS may not be appreciated despite difference in EFS and other measures. There can also be differences in histology (high incidence of embryonal component and rare seminomas) as well as more advanced disease at presentation for adolescents. Specifically, it appears that the incidence of clinical stage I disease decreases with age: 70–80% of prepubertal children with T-GCT, 50–60% of adolescents with T-GCT, and 40–50% of adults with NSGCT [24–27]. Another factor associated with age is marital status. Married AYAs with testicular cancer had improved OS and cancer-specific survival than their unmarried counterparts [17, 21].

### 2.4. Histology

Interestingly, T-GCTs in prepubertal males are usually pure yolk sac tumors, which rarely metastasize [28], and pure teratoma, which is benign in this age group [29]. Pure seminoma is rare in the pediatric and adolescent population. When compared to pure seminoma, nonseminoma is most common in the AYA population and generally is of mixed histology, more frequently involves metastatic disease at presentation, and has a higher rate of relapse [30]. These mixed tumors, especially with embryonal components, are the most common seen in AYAs [31].

Stokes et al. [32] recently performed an analysis of the NCDB looking at patterns of care and survival outcomes for AYAs (age ≥ 15 years) with seminoma treated with primary surgery, known histology, and known outcomes. They identified 12,880 AYAs and compared this group to both adults aged 40–55 years (8,022) and >55 years (1,459). Compared to their adult counterparts, AYAs in this cohort were more likely to be nonwhite/nonblack, be uninsured, have fewer comorbidities, have clinical stage 1 disease at presentation, receive care at a high-volume institution, forego RPLND, and undergo surveillance over adjuvant therapy. Unadjusted 5- and 10-year OS was significantly better for AYAs than their older counterparts (98% and 96.1%, resp.). Factors associated with improved OS included AYA age, private insurance, high facility volume, stage 1 disease, and receipt of radiation therapy. Even controlling for other factors, AYA status remains significantly associated with improved OS. Interestingly, race was not significantly associated with OS, unlike previous studies, while socioeconomic factors (insurance status) were associated with OS. The authors suggest that the less frequent use of adjuvant therapy by AYAs than older adults highlights progress for these patients; judicious use of these therapies, all of which carry significant side effects over the long term, has still allowed an extremely high OS [32]. This study mirrors findings from a SEER database analysis and European registry data [33].

A complementary paper on NSGCT in the AYA population investigated the SEER database to evaluate the association between age and outcomes [17]. The authors identified 1,496 adolescents (13–19 years) and 12,467 adults (>19 years) with a median follow up of 71 months. 5-year OS for adolescents was 94% and adults was 92% (*p*=0.007) with 5-year CSS of 95% and 94%, respectively (*p*=0.139). Age was a significant predictor of both OS and CSS when controlling for other factors. They also found that, despite presenting more often with metastatic disease, adolescents had improved OS and CSS than adults.

### 2.5. Risk Factors for Metastatic Disease

Active surveillance is the current recommendation for both adults and children [34] with clinical stage 1 T-GCTs. However, we know that a significant proportion (20–30%) will harbor occult disease. In the adult population, the identification of high-risk features for harboring of occult metastases—lymphovascular invasion and an increasing component of embryonal carcinoma for NSGCT [35] and size > 4 cm and rete testis invasion for seminoma [36]—has allowed a risk-stratified treatment approach to be employed [34]. Cost et al. [37] reviewed 23 patients aged 7–21 years and found that about half of all patients had high-risk features (≥40% embryonal carcinoma or lymphovascular invasion), and almost 60% with high-risk features harbored occult metastatic disease. No patients without high-risk features had metastatic disease. This confirmed that these same high-risk features for NSGCT in the adult population confer a similar risk for harboring occult metastatic disease in the pediatric and AYA population. While all relapses were successfully managed with 100% survival, the validation of these same high-risk features in the AYA population may lend themselves to counseling points for families and perhaps future incorporation into treatment strategies; however, they are currently not part of any treatment guidelines.

### 2.6. Surgery

Traditional teaching calls for radical orchiectomy for all testicular masses concerning for malignancy. Recent data suggest that partial orchiectomy/excisional biopsy via an inguinal incision may be safe in certain highly selected patients, and this has become common practice for the management of prepubertal pediatric testis tumors, regardless of the preoperative suspicion of teratoma [38]. For postpubertal boys, the authors' current practice involves performing a partial orchiectomy if patients have a mass < 2 cm *and* normal tumor markers, regardless of suspected pathology (manuscript in submission). Intraoperative frozen section is then utilized; if there is any concern for T-GCT, a radial orchiectomy is completed at the same setting. However, if the pathology returns benign or not concerning for T-GCT, the partial orchiectomy is completed and that testis has retained fertility and hormonal function [39]. Partial orchiectomy is not being advocated for or used to treat T-GCTs, but rather it is proposed as an initial step to preserve gonadal function in patients with small testicular masses and normal tumor markers due to the associated high rate of benign pathology. Although unilateral radical orchiectomy preserves contralateral testicular function, Leydig cell dysfunction and hypogonadism may develop prematurely, making T-GCT survivors at risk for androgen deficiency into adulthood [40].

### 2.7. Treatment

Because of similar tumor biology, postpubertal T-GCTs are best managed using adult algorithms. Individual pubertal status needs to be determined before discussing any treatment. Traditional pediatric regimens have been thought to undertreat adolescents with T-GCTs and may contribute to worse outcomes in adolescents over adults [6]. Indeed, the staging is different for patients with T-GCTs that are prepubertal (COG staging system) compared to postpubertal (AJCC TNMS system and IGCCCG system for metastatic disease), and the emphasis on postchemotherapy surgery differs. These differences are highlighted in Table 1. Additionally, COG remains concerned about long-term effects of cisplatin exposure (ototoxicity, nephrotoxicity, peripheral neuropathy, etc.) and is investigating the role of carboplatin versus cisplatin for children with T-GCTs. While adult studies have demonstrated a superior effect of cisplatin, pediatric studies have shown that higher dose carboplatin is associated with similarly good outcomes for children with T-GCTs [41]. Many adult urologic oncologists may be hesitant to place patients onto this COG study given their belief that randomization to the carboplatin arm is substandard of care therapy. COG protocols generally target patients aged 15 years and younger, with most postpubertal patients, which would include AYAs, being treated per adult algorithms [5].

The vast majority of adolescents and AYAs with clinical stage I disease should undergo active surveillance, per NCCN guidelines. The relapse rate is 20–30%, with excellent survival after salvage therapy. Even in the presence of high-risk features and high risk of relapse, the potential for morbidity with overtreatment of 70–80% of patients without a clear survival advantage makes an aggressive upfront treatment approach less desirable [42]. This approach prevents overtreatment and associated side effects while reserving highly effective salvage therapy for those who truly need it.

### 2.8. Long-Term Outcomes

There have been huge advances with long-term survival of AYAs with testicular cancer, so there has been a focus shift towards quality of life and late effects of treatment. A recent review of quality of life outcomes has shown that long-term testicular cancer survivors were comparable to age-matched controls, including mental health and sexual function, and that any decreases in quality of life were not related to treatment modality [43, 44]. For those treated during adolescence, however, lower rates of fertility, body image, and sexual function have been described [45].

## 3. Late Effects

Important to consider is that this group of patients has a longer life expectancy than older adults. Thus, the long-term sequelae of systemic treatments (radiation, chemotherapy, and surgery) should be seriously considered, and monitoring for these complications is necessary. The NCCN has published a clinical guideline for AYA oncology patients, which all providers caring for this group of patients should review and have readily available. This nicely summarizes risks specific to this patient population as well as screening guidelines for survivors [13].

### 3.1. Fertility

All adjunctive treatment strategies beyond radical orchiectomy (chemotherapy, RPLND, and radiation) are associated with potential fertility issues, either transient or permanent. A recent survey of cancer survivors ranked fertility questions as the second most common concern behind mortality [46]. Every effort should be made to perform nerve sparing RPLND when necessary, and some advocate for referral to high-volume centers. Sperm cryopreservation is the most effective method to maintain fertility potential, but this must be initiated prior to treatment for testicular cancer. There are a host of issues surrounding cryopreservation, including young age and collection methods, anxiety associated with cancer diagnosis, and high cost of preservation. AYA patients and their families may not immediately think of fertility to be important given a diagnosis of malignancy and the patient's current life stage, so it is the responsibility of the provider to address this issue head on, prior to treatment initiation. Early involvement of an oncofertility specialist can help patients and families work through banking [5].

### 3.2. Secondary Malignancy

For at least 35 years after treatment, patients who have received chemotherapy or radiation are at higher risk of developing a secondary malignancy over the general population who has not been exposed to these agents [47]. The relative risk of development of a secondary malignancy is 1.8 for radiation, 2 for chemotherapy, and 2.9 for a combination of chemotherapy and radiation [48]. Etoposide specifically carries a risk of developing a secondary leukemia that is highly resistant to available therapies. This risk is correlated with total dose received and is increased in combination with radiation exposure [49]. Smoking and excessive alcohol consumption, common behaviors in AYA cancer survivors, has been shown to increase the risk of malignancy in bladder/prostate rhabdomyosarcoma patients, who are also at increased risk of secondary malignancy due to the chemotherapeutic agents and radiation used to treat their disease [50]. Patients with T-GCTs may receive similar therapies to the rhabdomyosarcoma population, albeit with differing doses, fields, and agents. However, they too are at higher risk for secondary malignancies and probably also engage in cigarette use and excessive alcohol consumption. It is not unreasonable to infer that these patients may be further increasing their risk of malignancy with these behaviors and should be counseled to avoid these activities.

### 3.3. Chemotherapy

Cisplatin-related nephrotoxicity via proximal tubular dysfunction is well described. Decreases in glomerular filtration rate, hypomagnesemia, and proteinuria have all been reported with this drug that is highly effective for T-GCTs. A recent study calculated a 10% risk of stage 3 chronic kidney disease for those exposed to a median of 4 cycles of cisplatin during treatment, and rate of progression increased with more cycles of chemotherapy [51]. Another review of 63 children treated with cis- or carboplatin showed no significant change in renal function over time, measured 10 years after completion of therapy. However, 11% of patients had an eGFR < 60 mL/min/1.73 m^2^, which is not insignificant. Older age at the initiation of therapy was associated with a lower GFR [52].

About 1 in 6 patients will report peripheral neuropathy, and this is due to cisplatin exposure [53]. Similarly, high-frequency hearing loss is seen in 20–40% of patients exposed to cisplatin (dose-dependent), and this is usually permanent [54]. Other long-term effects of cisplatin exposure include cardiovascular disease, paresthesia, hypogonadism, hypercholesterolemia, and hypertension. Interestingly, studies have confirmed that platinum and platinum-based residuals remain in circulation up to 20 years, and it is thought that perhaps these contribute to long-term complications [55]. In this same study, renal function 1 year after treatment was associated with the level of platinum remaining, meaning that the relationship between renal function and drug goes both ways—the drug damages the kidneys, and because of this, there is more drug left in the system, perpetuating its effects [55]. It is postulated that there may be therapy-related vascular changes that could contribute to the increased cardiovascular disease and increased incidence (6%) of myocardial infarction in these patients, illustrating that the implication of a single agent/therapy as the cause for a specific complication has been difficult to determine thus far [56].

Bleomycin has been linked to lung disease in a dose-related fashion, with about 5% of patients developing pulmonary fibrosis. Risk factors for bleomycin toxicity include increased age, concomitant chest radiation, decreased renal function, and elevated concentrations of inspired oxygen. Unfortunately, radiographic evidence of bleomycin toxicity may be seen as pleural-based nodules, which may be mistaken for relapsed or refractory disease (these resolve over time) [54].

Metabolic syndrome has been reported to occur in about 25% of T-GCT survivors. The exact mechanism for this is unknown, but testosterone and Leydig cell function have been implicated, although not uniformly across studies [57].

Hypogonadism is estimated to occur in 10–15% of patients after unilateral orchiectomy [58, 59], resulting in the need for androgen replacement. Preserving gonadal function may also reduce the clinically underrecognized but real rates of osteopenia and osteoporosis in these patients [60].

### 3.4. Psychosocial Effects

Adolescence is a tumultuous time in life, where all changes and experiences are amplified. Most teenagers feel that even ordinary challenges are difficult to overcome and that they are facing these challenges alone. On top of this baseline feeling, a cancer diagnosis clearly radically changes the patient's life and their needs when confronted with cancer are greater than older patients. The AYA population has a significant need for psychosocial support; cancer and subsequent therapy will create significant change in their social lives and interactions, which are central to being a teenager. There are obvious changes that will occur; self-image will be affected by hair loss, weight changes, mood alterations, nausea, febrile illness and hospitalizations, isolation due to infectious risk, etc. Impaired sexual function due to infertility, impotence, and an inability to feel that the patient was having any type of intimate relationship are major issues during this life stage. While these issues are common for an adult urologist to discuss with their patients, regardless of whether the patient has cancer, pediatric patients and providers are often uncomfortable discussing these personal details. Conversely, adult providers rarely acknowledge the impact of adolescence and puberty on a patient's everyday health, attitudes, and compliance [32].

Patients in the AYA age group are often at a cross roads with respect to education and career decisions. Cancer obviously detracts from the attention that is usually paid to these decisions, which seriously impact a person's identity. Pursuing treatment may affect a patient's ability to work and earn an income, which may lead to financial challenges that are then augmented by the cost of cancer treatment and insurance issues that are already prevalent in this age group. In addition to the obvious financial implications, this may be associated with guilt about not being able to meet basic expectations. After therapy, in the survivorship stage, resuming normal work or school life activities can be difficult. More than half of cancer survivors have problems continuing work or education after therapy cessation [61]. Expectation for both the patient and employer/school is one of the biggest factors in the success of transitioning back to normal life. Additionally, maintaining some type of involvement in work/school life during therapy, even if minimal, is associated with increased success with reentry long term [61].

Relationships, both with friends and partners, are central to AYA lives and can be severely impacted by cancer diagnosis and treatment. Partners may be lost or have feelings of fear of relapse, guilt, or sympathy. A father or brother with testicular cancer increases a male's risk of testicular cancer four times over the general population (2 or more relatives, 10x increased risk), and the development of testicular cancer tends to be at a similar age, but not necessarily the same histology [62]. Thus, there are unknown genetics and predisposition for existing children and when considering expanding families, which may result in tension between partners and thus a strained relationship [5]. Additionally, care of young children during cancer treatment can be unpredictable and yet another source of strain on a relationship [13].

These challenges are more than those experienced in either the adult or pediatric population and thus providers are usually unprepared to handle them. Providers generally provide a narrow, focused, technical view of diagnosis and treatment, which may further isolate the patient and his family, marginalizing their concerns. To fix this, early involvement of a multidisciplinary team, including mental health providers, is necessary. Not only will this improve mental health, stress levels, and quality of life, but it will increase compliance and hopefully survival, a central issue with the AYA population. Being aware of the issues, creating a team, and being prepared is the first step to face these issues head on [5].

## 4. Conclusions and Future Directions

For all the above reasons, the AYA population truly is unique with its own particular set of challenges. While the end goal is to improve outcomes, namely survivorship, there are a host of other issues that need to be addressed. These issues will not be able to be tackled without a multilayered approach to both clinical and translational research. AYA oncology education and awareness need to be increased, areas of research that will most directly lead to improved survivorship or quality of life for need to be prioritized, and there needs to be increased funding for researchers committed to studying this population. Increased awareness on the national level with various new societies and groups is occurring, but urologists need to be advocates at the institutional level to raise awareness and education about this unique population [5].

Recently, novel biomarkers such as microRNA clusters have been identified that are uniformly overexpressed in all malignant GCTs, regardless of patient age, subtype, or site. While these remain a research tool and are not yet prevalent in everyday practice, they remain an exciting possibility for diagnosis (new staging criteria?) and surveillance (instead of CT scans?) of patients with T-GCTs [63].

Patient care collaboration through the development of and referral to highly experienced treatment teams have been shown to improve outcome for patients with T-GCTs. With increasing technology available to share information between centers, expertise can reach farther than a single institution into smaller community practices for advice and allows for improved coordinated referrals to these large volume centers [64]. A huge area in need of improvement for this group is clinical trial participation. More than 90% of children participate in clinical trials, while about 10% of teenagers and even fewer young adults do participate [28]. Providers need to educate patients and families about study trial opportunities that exist and need to create trials that specifically target this population [5].

## Figures and Tables

**Table 1:** AJCC versus COG staging for testicular tumors [5].

Stage	AJCC	COG
I	pT_1-4_N_0_M_0_S_0_ IA: pT_1_N_0_M_0_S_0_ IB: pT_2-4_N_0_M_0_S_0_ IS: pT_1-4_N_0_M_0_S_1-3_	Tumor limited to testis, completely resected by high inguinal orchiectomy; no clinical, radiographic, or histologic evidence of disease beyond the testis Normal or unknown tumor markers at diagnosis must have negative ipsilateral RPLND to confirm stage I disease if imaging shows LNs > 2 cm Scrotal orchiectomy with high ligation of the cord is also considered stage I
II	pT_1-4_N_1-3_M_0_S_0-1_ IIA: pT_1-4_N_1_M_0_S_0-1_ IIB: pT_1-4_N_2_M_0_S_0-1_ IIC: pT_1-4_N_3_M_0_S_0-1_	Transscrotal biopsy; microscopic disease in scrotum or high in spermatic cord (< 5 mm from proximal cord margin) Failure of tumor markers to normalize or decrease with an appropriate half-life
III	pT_1-4_N_1-3_M_1_S_0-1_ IIIA: pT_1-4_N_1-3_M_1a_S_0-1_ IIIB: pT_1-4_N_1-3_M_0-1b_S_2_ IIIC: pT_1-4_N_1-3_M_0-1b_S_3_	Retroperitoneal LN involvement without visceral or extraabdominal involvement LNs > 4 cm by CT or 2–4 cm if biopsy proven metastatic
IV		Distant metastasis, including liver

## Abbreviations

OS: Overall survival
AYA: Adolescent and young adult
NCCN: National Comprehensive Cancer Network
NCI: National Cancer Institute
CSS: Cancer-specific survival
T-GCT: Testicular germ cell tumor
NSGCT: Nonseminomatous germ cell tumor
EFS: Event free survival
NCDB: National Cancer Database
RPLND: Retroperitoneal lymph node dissection
SEER: Surveillance, Epidemiology and End Results Program
COG: Children's Oncology Group
SWOG: Southwest Oncology Group
AJCC: American Joint Committee on Cancer
TNMS: Tumor, node, metastasis, serum
IGCCCG: International Germ Cell Cancer Collaborative Group
eGFR: Estimated glomerular filtration rate.
